# Neurological complications of sepsis

**DOI:** 10.1097/MCC.0000000000001022

**Published:** 2023-02-15

**Authors:** Simone Piva, Michele Bertoni, Nicola Gitti, Francesco A. Rasulo, Nicola Latronico

**Affiliations:** a Department of Medical and Surgical Specialties, Radiological Sciences and Public Health, University of Brescia; b Department of Anesthesia, Critical Care and Emergency, Spedali Civili University Hospital; c ’Alessandra Bono’ University Research Center on Long-term Outcome in Critical Illness Survivors, University of Brescia, Brescia, Italy

**Keywords:** delirium, sepsis, sepsis-associated encephalopathy

## Abstract

Sepsis, defined as life-threatening organ dysfunction caused by a dysregulated host response to infection, is a leading cause of hospital and ICU admission. The central and peripheral nervous system may be the first organ system to show signs of dysfunction, leading to clinical manifestations such as sepsis-associated encephalopathy (SAE) with delirium or coma and ICU-acquired weakness (ICUAW). In the current review, we want to highlight developing insights into the epidemiology, diagnosis, prognosis, and treatment of patients with SAE and ICUAW.

**Recent findings:**

The diagnosis of neurological complications of sepsis remains clinical, although the use of electroencephalography and electromyography can support the diagnosis, especially in noncollaborative patients, and can help in defining disease severity. Moreover, recent studies suggest new insights into the long-term effects associated with SAE and ICUAW, highlighting the need for effective prevention and treatment.

**Summary:**

In this manuscript, we provide an overview of recent insights and developments in the prevention, diagnosis, and treatment of patients with SAE and ICUAW.

## INTRODUCTION

Sepsis, defined as life-threatening organ dysfunction caused by a dysregulated host response to infection [1], is a leading cause of hospital and ICU admission. Sepsis-associated mortality remains high among critically ill patients [2], and early recognition and treatment of sepsis are of vital importance to reduce mortality [3]. The nervous system may be the first organ to show signs of dysfunction, especially in elderly and immunocompromised patients, leading to a wide range of clinical syndromes, including sepsis-associated encephalopathy (SAE), seizures, cerebrovascular events, and neuromuscular disorders that increase mortality and ICU length of stay (LOS).

SAE is a diffuse cerebral dysfunction originating from a systemic inflammatory response to sepsis. Its clinical manifestations range from mild delirium to severe coma and are associated with an increased mortality rate [4] and long-term physical, mental and cognitive dysfunctions [5].

The systemic inflammatory responses triggered during sepsis can also affect the peripheral nerves, skeletal muscles, or both, ultimately leading to critical illness polyneuropathy (CIP) and myopathy (CIM), along with disuse atrophy. This pathological sequence sets the base for what is known as ICU-acquired weakness, which affects 25% of prolonged mechanically ventilated patients, and up to 65% of septic patients. It causes difficulty in weaning from the ventilator, prolonged ICU stay, and increases long-term morbidity and mortality [6,7].

This review aims to clarify key aspects of the neurological sequelae of sepsis, providing useful clinical information on the diagnosis, management, and prognosis. 

**Box 1:**
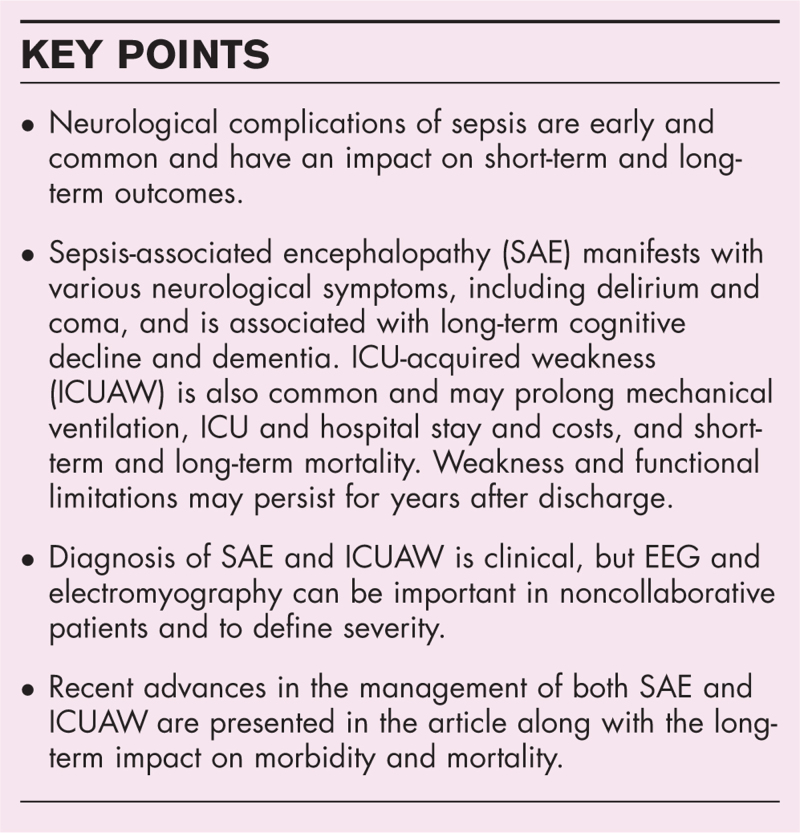
no caption available

## SEPSIS-ASSOCIATED ENCEPHALOPATHY

### Definition

SAE is the most common neurologic complication of sepsis. SAE can be defined as a condition of acute encephalopathy [8] arising in patients with clinical evidence of sepsis or septic shock [9^▪^], which cannot be attributed to drug intoxication, fat embolism syndrome, autoimmune or inflammatory brain diseases, such as vasculitis or thrombotic microangiopathy, acute brain infections such as meningitis or encephalitis, anoxic brain injury, or other acute primary brain diseases [10–16]. Thus, SAE is diagnosed by exclusion, as the acute encephalopathy should not be attributable to any other cause than sepsis itself. SAE is usually described as an organ dysfunction caused by a dysregulated host response without direct infection of the brain. However, postmortem neuropathology studies have reported cerebral abscesses in 10% of patients with septic shock. Moreover, viable gut-associated bacteria have been demonstrated in mice brains 5 days after surviving experimental peritonitis [17].

SAE manifests as a rapid change from baseline cognitive status or level of consciousness, and consists of a wide range of symptoms, from subsyndromal delirium to coma [8]. Lacking a clear consensus on the definition, a precise description of the epidemiological features of SAE is challenging: reported incidence varies from 9 to 71% [10,12,18]. In a recent retrospective study [19], the incidence of SAE in a cohort of 291 septic patients was 43.6%. Although SAE is a reversible syndrome, mild-to-moderate residual neuropsychiatric symptoms including depression, anxiety, or cognitive disturbances, may persist in up to 40% of patients 1 year after hospital discharge [20,21].

The pathophysiology of SAE is multifactorial and the exact molecular and cellular alterations are still poorly understood. The mechanisms underlying SAE include a complex interaction between systemic inflammation and cytokine storm derived from sepsis, endothelial damage with loss of blood–brain barrier integrity, neuroinflammation and glial activation, altered neuronal metabolism, oxidative stress, and impaired brain perfusion. Multifactorial cellular damage is likely to result in a profound alteration in brain electrochemical signaling of varying magnitude, clinically manifested as delirium or coma in the most severe cases [22].

### Diagnosis

The diagnosis of SAE is clinical and one of exclusion when septic patients develop clinical signs of an acute encephalopathy with or without focal neurological deficit, in the absence of other neurological, systemic, or metabolic conditions that may explain the acute encephalopathy [18].

Clinical manifestations of SAE encompass impairment of attention, cognition, and consciousness, ranging from delirium (50%) to coma (46%) [22,23]. Delirium in SAE is more frequently hypoactive than hyperactive, and it can be associated with focal deficits, seizures, asterixis, or tremors [22].

As SAE is often the first sign of organ failure to occur in sepsis, physicians must look for sepsis in any patient who develops delirium [22,24]. Indeed, clinical neurological screening should be done using validated tools such as the Confusion Assessment Methods for the Intensive Care Unit [25], the Intensive Care Delirium Screening Checklist [26], the Glasgow Coma Scale, and the Full Outline of UnResponsiveness (FOUR) Score [25].

An accurate differential diagnosis of SAE is often complicated by systemic disturbances, including multiorgan failure, hypoglycemia, electrolyte abnormalities, or severe hypoxemia, conditions that can cause acute nonseptic-related acute encephalopathy. Moreover, septic patients may share many of the predisposing and precipitating risk factors for nonsepsis-associated delirium, such as advanced age, frailty, baseline cognitive impairment, metabolic disturbances, sedation, hypotension, multiple comorbidities, sleep disturbances, and surgery [27,28].

Electroencephalography (EEG) and neuroimaging may help in the differential diagnosis of SAE [11,16].

Although EEG patterns described in SAE are not specific, they occur in a majority of patients with sepsis and are associated with the severity of SAE. EEG abnormalities include generalized slowing in the background activity, and the presence of theta and delta waves, which are indicators of diffuse cortical dysfunction [29]. Theta waves often appear in patients with mild and moderate encephalopathy (confusion, delirium). Delta activity appears in a coma, where the impairment in consciousness level is more severe and during deep sedation [30^▪^]. The presence of triphasic waves and a burst-suppression pattern indicates the dysfunction of deeper brain structures [11]. Young's classification provides a structured approach and defines how these data should be interpreted [31]. Interestingly, mortality is increased in patients with severe abnormalities on EEG; patients who have triphasic waves or burst-suppression patterns on EEG have higher mortality rates than those with abnormal theta or delta wave patterns [31,32]. Lastly, sepsis can be associated with electroencephalographic seizures or periodic epileptiform discharges [33].

Neuroimaging techniques, such as CT scans and MRI, are often unremarkable in patients with SAE. Indeed, a CT scan should be performed in patients with SAE to exclude brain lesions or intracranial abnormalities, but brain lesions can be detected in the most severe patients and are related to disease severity [34]. MRI abnormalities are present in up to 60% of patients with SAE [35,36], with heterogeneous and nonspecific patterns including ischemic lesions [36], leukoencephalopathy [35,36], vasogenic edema with signs of posterior reversible encephalopathy syndrome possibly attributable to blood–brain barrier breakdown [37], and white matter hyperintensity in T2-weighted image [38,39].

### Management

The cornerstone of SAE management relies on early diagnosis and prompt treatment of infection and organ dysfunction, including states of mildly altered consciousness and delirium.

Severe sepsis in the elderly population is independently associated with a tripling in the odds of moderate-to-severe new cognitive impairment [40]. Septic episodes are associated with the development of dementia within 10 years independent of age and other risk factors [41]. This suggests that effective sepsis prevention and treatment may reduce the risk of long-lasting, profound cognitive impairment in survivors.

Reduced consciousness is associated with a reduced ability to clear tracheobronchial secretions, increases the risk of pulmonary complications, and requires strict patient surveillance and supportive care. To date, there are no evidence-based pharmacological options that have demonstrated efficacy in the prevention and/or treatment of delirium in SAE. The PADIS guidelines [42] advise against routine usage of haloperidol, atypical antipsychotics, or statins to treat delirium. If antipsychotics are chosen to manage the hyperactive behavior of delirious patients, or stress-related symptoms (anxiety, hallucinations, delusion, fear, etc.), they should be used in the lowest dose and for the shortest period possible. In a recent, large randomized controlled trial in ICU patients with delirium, treatment with haloperidol did not significantly increase the number of days alive and out of the hospital at 90 days compared with placebo [43]. Melatonin did not reduce the prevalence of delirium when administered prophylactically in a large randomized controlled trial of 847 patients, of whom a quarter had sepsis as an admission diagnosis [44]. A recent systematic review and meta-analysis of 77 trials constituting 11 997 critically ill patients found that in mechanically ventilated adults, the use of dexmedetomidine compared with other sedatives resulted in a lower risk of delirium, and a modest reduction in the duration of mechanical ventilation and ICU stay at a cost of increasing the risk of bradycardia and hypotension [45^▪▪^]. Prophylactic use of antiepileptic drugs is not recommended, and EEG monitoring should be used in comatose or deeply sedated patients to detect nonconvulsive seizures and guide therapy [16].

Multimodal interventions such as the ABCDEF bundle (A assess, prevent, and manage pain; B both spontaneous awakening and spontaneous breathing trials; C choice of analgesic and sedation; D delirium: assess, prevent, and manage; E early mobility, and exercise; and F family engagement and empowerment) have been shown to be effective in improving patient outcomes. Implementation of such a bundle may increase days alive and freedom from delirium and coma [46], lower the likelihood of death within 7 days, lower physical restraint use, lower ICU readmissions, lower discharge to a facility other than home [47], and decrease ICU and hospital length of stay [48].

### Prognosis

Beyond short-term life-threatening complications, adult sepsis survivors experience increased long-term mortality and morbidity, higher rates of rehospitalization, and reduced quality of life. Mortality rates after surviving the initial sepsis episode remain high; the 1-year postdischarge mortality rate varies between 7 and 43%, depending on sepsis severity [49] and age [50]. Long-term mortality and morbidity are often because of the ‘postsepsis syndrome’, a condition characterized by an increased risk of developing physical, cognitive, and mental health problems after sepsis [5,51].

Duration of delirium in septic patients is strongly associated with the development of cognitive impairment and increased odds of disability in daily life activities at long-term follow-up [52]. Therefore, monitoring of delirium during the acute stage of the disease may provide important clues to the risk of subsequent cognitive impairment. Markers of systemic inflammation and coagulation measured early in the ICU are not associated with long-term cognitive outcomes [53].

## ICU-ACQUIRED WEAKNESS

Systemic inflammation during sepsis can negatively impact peripheral nerves, limb skeletal muscle, and the diaphragm. ICUAW, along with diaphragmatic weakness, is the most common neuromuscular impairment in ICU patients, and it is detected in up to 67% of patients with sepsis [6]; diaphragmatic weakness seems to develop earlier than limb weakness and is more frequent [54–56].

### Definition

**FIGURE 1:**
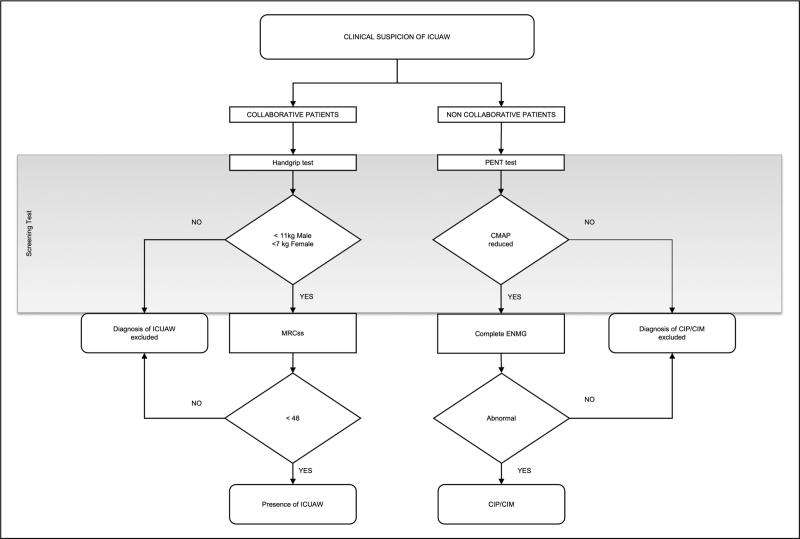
Diagnostic flow-chart for ICU-acquired weakness (ICUAW), critical illness polyneuropathy, and critical illness myopathy. CMAP, compound muscle action potential; ENMG, electroneuromyography; MRCss, Medical Research Council Sum Score; PENT, peroneal nerve test.

ICUAW is defined as a symmetrical weakness that arises after the onset of a critical illness, affecting all four limbs and the respiratory muscles with sparing of the facial muscles. The muscle tone is reduced, but the deep tendon reflex can either be reduced or normal. ICUAW is due to a different grade of overlap between the critical illness polyneuropathy (CIP), critical illness myopathy (CIM), and muscle disuse atrophy. [57] The simultaneous presence of CIP and CIM or critical illness polyneuromyopathy is the most frequent condition [58]. CIP is defined as a sensory–motor axonal polyneuropathy (Fig. 1), whereas CIM is an acute primary myopathy, not related to denervation.

### Diagnosis

The flowchart of ICUAW diagnosis (Fig. 1 and Table 1) is based on the patient's level of consciousness.

In awake, collaborative patients, the diagnosis is clinical and relies on the Medical Research Council Sum Score (MRCss), in which the strength of functional muscle groups of the limbs is graded from 0 to 5 (i.e. none, weak, poor, acceptable, good, and normal) [59]. The scores obtained from the six bilateral muscle groups (wrist flexion, forearm flexion, shoulder abduction, ankle dorsiflexion, knee extension, and hip flexion) can range from 0 (complete paralysis) to 60 (normal muscle strength). A clinically relevant ICUAW is defined as MRCss below 48/60, whereas severe ICUAW is defined as below 36/60 [59,60]. A four-point ordinal MRCss scale has been recently introduced, but validation is still pending [61]. Although the inter-rater reliability of the MRCss remains high (intraclass correlation coefficient from 0.83 [62] to 0.99 [63]), its correct application requires specific training and is time-consuming. Handgrip dynamometry (HGD) is a simple, inexpensive, and repeatable test that can be used as a screening test for ICUAW. Although the HGD values depend on sex and age, an absolute cut-off value of 11 kg in men and 7 kg in women allows discrimination of the ICUAW presence with a sensitivity of 0.81 and a specificity of 0.83 [64,65].

**FIGURE 2:**
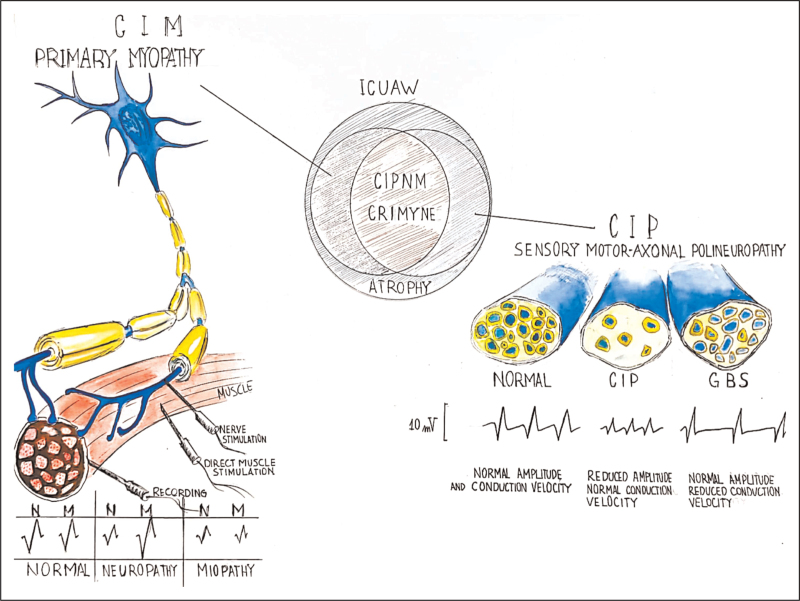
Differential diagnosis of critical illness polyneuropathy and critical illness myopathy. Critical illness polyneuropathy (CIP) is an acute sensory–motor axonal neuropathy with reduced nerve action potential amplitude and normal conduction velocity. In demyelinating Guillain–Barré syndrome, the nerve action potential amplitude is normal while the nerve conduction velocity is reduced (right). Direct muscle stimulation (DMS) may distinguish CIP from CIM in noncollaborative patients. In CIM, the compound muscle action potentials (CMAPs) obtained through nerve stimulation (neCMAP) and direct muscle stimulation (dmCMAP) are proportionally reduced and their ratio is around 1. In CIP, the CMAP is reduced, while dmCMAP is normal and their ratio is small and around zero (left). However, CIP and CIM often coexist in patients with ICUAW (center). Adapted from Latronico *et al. Curr Opin Crit Care* 2005; 11: 126; and Kramer *et al. Neurol Clin* 2017; 35: 723. CIM, critical illness myopathy; CIP, critical illness polyneuropathy; CIPNM, critical illness polyneuromyopathy; CMAP, compound muscle action potential; CRIMYNE, critical illness myopathy and neuropathy; DMS, direct muscle stimulation; GBS, Guillain–Barré syndrome; ICUAW, ICU-acquired weakness.

In noncollaborative patients, the presence of CIM and CIP can be established using electrophysiology (Fig. 2 and Table 1). CIP is an axonal sensorimotor polyneuropathy depicting a reduction in the total number of nerve fibers. In nerve conduction studies (NCS), this is reflected as reduced amplitudes on compound motor action potential (CMAP), sensory nerve action potential (SNAP), or both. The myelin sheath is not affected in CIP, indeed NCS shows normal velocity and normal latency. This feature is an important factor in differentiating between CIP and Guillain–Barré syndrome. A simplified screening test (peroneal nerve test, PENT) has been proposed that solely evaluates the CMAP amplitude of the peroneal nerves. A PENT with CMAP amplitude below a normal value had 100% sensitivity and high specificity in diagnosing CIP/CIM [54,66].

CIM is a primary myopathy, and needle electromyography (EMG) examination in collaborative ICU patients performing voluntary muscle contraction shows short-duration, low-amplitude, polyphasic motor unit potentials with early or normal full recruitment together with a normal SNAP. Fibrillation potentials at rest indicate nonspecific changes in the muscle arising either from the nerve or directly from the muscle. Patients in the ICU may not be cooperative enough to evaluate the morphological aspects of the motor units and the recruitment patterns, two pivotal factors in differentiating CIP from CIM. For this reason, direct muscle stimulation (DMS) in conjunction with standard nerve testing may help in distinguishing CIP from CIM in noncollaborative patients. With DMS, both the stimulating and recording electrodes are placed over the muscle belly. A patient with CIM will have proportionally reduced CMAP amplitude after both standard stimulation and DMS. The ratio between the CMAP obtained through nerve stimulation and the CMAP obtained through DMS (neCMAP/dmCMAP) will be around 1 and the dmCMAP will be reduced below 3 mV (normal values are above 3.0–3.2 mV) [57]. Conversely, in a patient with CIP, the CMAP will be reduced, whereas dmCMAP will be normal and their ratio will be below small and around zero (Fig. 2 and Table 1) [67,68].

**Table 1:** Diagnostic criteria for ICU-acquired weakness, critical illness polyneuropathy, and critical illness myopathy

	ICUAW	
Clinical diagnosis [69]	Minimum criteria for diagnosing ICUAW: 1, 2, 3 and 5 or 4 and 5 (1) Generalized weakness developing after the onset of critical illness (2) Weakness is diffuse (involving both proximal and distal muscles), symmetric, flaccid, and generally spares cranial nerves (3) MRC sum score less than 48, or mean MRC sum score less than 4 in all testable muscle groups noted on at least two occasions separated by 24 h (4) Dependence on mechanical ventilation (5) Causes of weakness not related to the underlying critical illness have been excluded	
	CIP	CIM
Electrophysiological diagnosis [58]	Definite CIP: all of the following criteria Probable CIP: criteria 1, 3, 4 and 5 (1) The patient is critically ill (2) Diagnosis of ICUAW established (3) CMAP amplitudes less than 80% of the lower limit of normal in at least two motor nerves (4) SNAP amplitudes less than 80% of the lower limit of normal in at least two sensory nerves (5) Absence of a decremental response on repetitive nerve stimulation	Definite CIM: all of the following criteria Probable CIM: criteria 1 and 3–6 (1) The patient is critically ill (2) Diagnosis of ICUAW established (3) CMAP amplitudes less than 80% of the lower limit of normal in at least two nerves (4) SNAP amplitudes normal (5) EMG with short duration, low-amplitude MU potentials with early or normal full recruitment, with or without fibrillation potentials in collaborative patients; or reduced muscle action potential amplitude less than 3 mV and muscle/nerve action potential amplitude around 1 on DMS in noncollaborative patient (6) Absence of a decremental response on repetitive nerve stimulation (7) Muscle histology consistent with myopathy
Muscle biopsy [58,70]	Features of denervation and reinnervation with small muscle fibers, fiber-type grouping, and fiber group atrophy	Thick filament myopathy with a selective loss of myosin filaments; muscle necrosis; acute diffuse necrotizing myopathy.
Nerve biopsy [58,70,71]	Widespread axonal degeneration of both motor and sensory nerves.	Normal

CIM, critical illness myopathy; CIP, critical illness polyneuropathy; CMAP, compound muscle action potential; DMS, direct muscle stimulation; EMG, electromyogram; ICUAW, ICU-acquired weakness; MRC sum score, Medical Research Council sum score; MUP, motor unit potential; SNAP, sensory nerve action potential.

Although electrophysiological abnormalities are useful to establish the presence of CIP and/or CIM, their presence does not invariably correlate with clinically detectable muscle weakness. Indeed, pure electrophysiological alterations are clinically relevant, as they predict functional limitations at ICU discharge [72] and increased long-term mortality 1–5 years after ICU or hospital discharge [73,74].

### Management

As there are still no specific therapies with proven benefits on ICUAW, it is crucial to aggressively treat sepsis and implement strategies to minimize exposure to ICUAW risk factors.

Light sedation and early mobilization are probably the most effective strategies to prevent ICUAW. Enacting program to reduce sedatives to the minimal dose possible for patient comfort and safety and implementing early rehabilitation maneuvers and occupational therapy in the ICU may be an effective strategy to avoid immobility and prevent ICUAW [75]. Several studies showed that early mobilization may be feasible and well tolerated, may improve physical function, decrease the risk of ICUAW and delirium, and shorten the time to weaning from mechanical ventilation [76–80]. However, other studies have shown contrasting results [81–83] and, therefore, the efficacy of early mobilization remains uncertain [84^▪^]. Moreover, a strategy to achieve the maximum intensity of mobilization tolerated by patients at an early stage should be avoided, as it does not improve short-term outcomes and is associated with increased adverse events when compared with a lower level of early mobilization [85^▪▪^]. Instead, a step-by-step increase in the level of early mobilization adjusted to the patient's medical situation, muscle strength, and level of cooperation should be pursued.

Hyperglycemia should be promptly treated. Although normalizing glycemia reduces the electrophysiological sign of CIP/CIM and the need for mechanical ventilation [86,87], mortality is increased in patients treated with insulin to achieve normoglycemia as compared with patients receiving insulin to target blood glucose below 180 mg/dl [88]. The optimal blood glucose target still needs to be established [89].

The old paradigm that full caloric and protein intakes should be implemented early has been challenged by studies reporting a more pronounced muscle wasting associated with increased protein delivery during the first week in the ICU [90]. Indeed, no clear evidence supports amino acid supplements [91], and a post hoc analysis of EPaNIC (Early Parenteral Nutrition to Supplement Insufficient Enteral Nutrition in Intensive Care trial) confirmed a higher incidence of ICUAW in early parenteral nutrition patients [92]. These findings suggest that the early catabolic phase during critical illness cannot be averted by artificial nutrition, as critically ill patients are not able to use the exogenous amino acids for muscle protein synthesis [93^▪▪^].

### Prognosis

CIM and CIP have a decisive influence on the prognosis of intensive care patients. ICUAW is associated with difficult liberation from mechanical ventilation, higher extubation failure rate, postextubation dysphagia, impaired effective cough, prolonged ICU and hospital stay, increased hospital cost, and increased short-term mortality. In the long-term, ICUAW is associated with increased weakness, reduced walking exercise ability, reduced quality of life, and increased long-term mortality [7,89].

Recovery from weakness typically occurs within weeks or months [94], but the most severe cases may not recover, with many ICU survivors continuing to demonstrate weakness and persistent functional impairments (e.g. mobility, coordination, self-care, endurance). In two studies, the long-term sequelae of ICUAW have been reported for up to 5 years [95,96]. Other studies also describe persistent physical disability [97–99]. There are myriad contributors to the persistence of reduced muscle and strength, and diminished exercise capacity after sepsis [97], and include the severity of the ICUAW and underlying cause. The multicentre Italian study CRIMYNE (CRitical Illness MYopathy and/or Neuropathy) found that CIM has a better prognosis than CIP [100–102]. Physical impairment substantially impacts the quality of life [74,97,98,103] and joblessness [95,102,104,105]. Moreover, survivors who do not return to work report worse health-related quality of life compared with patients returning to work, thus creating a vicious cycle [104].

## CONCLUSION

With an estimated 50 million new cases reported every year [106], sepsis is a major health problem worldwide. Neurological complications such as acute encephalopathy, delirium, coma, reduced mobility, exercise tolerance, weakness, neuropathy, and myopathy have a profound impact not only on mortality but also on persistent morbidity and reduced quality of life in survivors. Rapid recognition of neurological complications is vital and can be supported by the use of ENG-EMG and EEG, but treatments remain supportive. We urgently need mechanistic studies to understand the pathophysiology of these complications and to develop more specific treatments. Combining treatments, such as early mobilization, personalized use of sedative drugs, cognitive stimulation, and appropriate nutritional strategy would be important to reduce the burden of cognitive and physical disability in sepsis survivors.

## Acknowledgements

*The authors would like to thank Vitaliy Kharchuk (nurse) for his contributions in*Fig. 2.

### Financial support and sponsorship

*None.*

### Conflicts of interest

*There are no conflicts of interest.*

## REFERENCES AND RECOMMENDED READING

Papers of particular interest, published within the annual period of review, have been highlighted as:

▪ of special interest
▪▪ of outstanding interest

## References

[1] Singer M Deutschman CS Seymour CW. The Third International Consensus Definitions for Sepsis and Septic Shock (Sepsis-3). JAMA 2016; 315:801–810. 26903338 10.1001/jama.2016.0287 PMC4968574

[2] Angus DC Linde-Zwirble WT Lidicker J. Epidemiology of severe sepsis in the United States: analysis of incidence, outcome, and associated costs of care. Crit Care Med 2001; 29:1303–1310. 11445675 10.1097/00003246-200107000-00002

[3] Seymour CW Gesten F Prescott HC. Time to treatment and mortality during mandated emergency care for sepsis. N Engl J Med 2017; 376:2235–2244. 28528569 10.1056/NEJMoa1703058 PMC5538258

[4] Sonneville R de Montmollin E Poujade J. Potentially modifiable factors contributing to sepsis-associated encephalopathy. Intensive Care Med 2017; 43:1075–1084. 28466149 10.1007/s00134-017-4807-z

[5] Mostel Z Perl A Marck M. Postsepsis syndrome - an evolving entity that afflicts survivors of sepsis. Mol Med 2019; 26:6. 31892321 10.1186/s10020-019-0132-z PMC6938630

[6] Fan E Cheek F Chlan L. An official American Thoracic Society Clinical Practice guideline: the diagnosis of intensive care unit-acquired weakness in adults. Am J Respir Crit Care Med 2014; 190:1437–1446. 25496103 10.1164/rccm.201411-2011ST

[7] Latronico N Herridge M Hopkins RO. The ICM research agenda on intensive care unit-acquired weakness. Intensive Care Med 2017; 43:1270–1281. 28289812 10.1007/s00134-017-4757-5

[8] Slooter AJC Otte WM Devlin JW. Updated nomenclature of delirium and acute encephalopathy: statement of ten Societies. Intensive Care Med 2020; 46:1020–1022. 32055887 10.1007/s00134-019-05907-4 PMC7210231

[9▪] Evans L Rhodes A Alhazzani W. Surviving sepsis campaign: international guidelines for management of sepsis and septic shock 2021. Intensive Care Med 2021; 47:1181–1247. 34599691 10.1007/s00134-021-06506-y PMC8486643

[10] Gofton TE Young GB. Sepsis-associated encephalopathy. Nat Rev Neurol 2012; 8:557–566. 22986430 10.1038/nrneurol.2012.183

[11] Hosokawa K Gaspard N Su F. Clinical neurophysiological assessment of sepsis-associated brain dysfunction: a systematic review. Crit Care 2014; 18:674. 25482125 10.1186/s13054-014-0674-y PMC4277650

[12] Young GB Bolton CF Austin TW. The encephalopathy associated with septic illness. Clin Invest Med 1990; 13:297–304. 2078909

[13] Davies NWS Sharief MK Howard RS. Infection-associated encephalopathies—their investigation, diagnosis, and treatment. J Neurol 2006; 253:833–845. 16715200 10.1007/s00415-006-0092-4

[14] Oldham MA Holloway RG. Delirium disorder. Neurology 2020; 95:173–178. 32518149 10.1212/WNL.0000000000009949

[15] Ebersoldt M Sharshar T Annane D. Sepsis-associated delirium. Intensive Care Med 2007; 33:941–950. 17410344 10.1007/s00134-007-0622-2

[16] Robba C Crippa IA Taccone FS. Septic encephalopathy. Curr Neurol Neurosci Rep 2018; 18:82. 30280261 10.1007/s11910-018-0895-6

[17] Singer BH Dickson RP Denstaedt SJ. Bacterial dissemination to the brain in sepsis. Am J Respir Crit Care Med 2018; 197:747–756. 29232157 10.1164/rccm.201708-1559OC PMC5855074

[18] Ely EW Shintani A Truman B. Delirium as a predictor of mortality in mechanically ventilated patients in the intensive care unit. JAMA 2004; 291:1753–1762. 15082703 10.1001/jama.291.14.1753

[19] Chen J Shi X Diao M. A retrospective study of sepsis-associated encephalopathy: epidemiology, clinical features and adverse outcomes. BMC Emerg Med 2020; 20:77. 33023479 10.1186/s12873-020-00374-3 PMC7539509

[20] Barichello T Sayana P Giridharan VV. Long-term cognitive outcomes after sepsis: a translational systematic review. Mol Neurobiol 2019; 56:186–251. 29687346 10.1007/s12035-018-1048-2

[21] Semmler A Widmann CN Okulla T. Persistent cognitive impairment, hippocampal atrophy and EEG changes in sepsis survivors. J Neurol Neurosurg Psychiatry 2013; 84:62–69. 23134661 10.1136/jnnp-2012-302883

[22] Mazeraud A Righy C Bouchereau E. Septic-associated encephalopathy: a comprehensive review. Neurotherapeutics 2020; 17:392–403. 32378026 10.1007/s13311-020-00862-1 PMC7283452

[23] Heming N Mazeraud A Verdonk F. Neuroanatomy of sepsis-associated encephalopathy. Crit Care 2017; 21:1–6. 28320461 10.1186/s13054-017-1643-z PMC5360026

[24] Rasulo FA Bellelli G Ely EW. Are you Ernest Shackleton, the polar explorer? Refining the criteria for delirium and brain dysfunction in sepsis. J Intensive Care Med 2017; 5:23. 10.1186/s40560-017-0218-z PMC5341444 28286656

[25] Sharshar T Citerio G Andrews PJD. Neurological examination of critically ill patients: a pragmatic approach. Report of an ESICM expert panel. Intensive Care Med 2014; 40:484–495. 24522878 10.1007/s00134-014-3214-y

[26] Gusmao-Flores D Salluh JIF Chalhub RÁ Quarantini LC. The confusion assessment method for the intensive care unit (CAM-ICU) and intensive care delirium screening checklist (ICDSC) for the diagnosis of delirium: a systematic review and meta-analysis of clinical studies. Crit Care 2012; 16:R115. 22759376 10.1186/cc11407 PMC3580690

[27] Hayhurst CJ Pandharipande PP Hughes CG. Intensive care unit delirium: a review of diagnosis, prevention, and treatment. Anesthesiology 2016; 125:1229–1241. 27748656 10.1097/ALN.0000000000001378 PMC5119532

[28] Kotfis K Marra A Ely EW. ICU delirium - a diagnostic and therapeutic challenge in the intensive care unit. Anaesthesiol Intensive Ther 2018; 50:160–167. 29882581 10.5603/AIT.a2018.0011

[29] Guérit J-M. Neurophysiological testing in neurocritical care. Curr Opin Crit Care 2010; 16:98–104. 20168224 10.1097/MCC.0b013e328337541a

[30▪] Rasulo FA Hopkins P Lobo FA. Processed electroencephalogram-based monitoring to guide sedation in critically ill adult patients: recommendations from an international expert panel-based consensus. Neurocrit Care 2022; [Epub ahead of print]. 10.1007/s12028-022-01565-5 PMC10090014 35896766

[31] Young GB Bryan Young G Bolton CF. The electroencephalogram in sepsis-associated encephalopathy. J Clin Neurophysiol 1992; 9:145–152. 1552002 10.1097/00004691-199201000-00016

[32] Oddo M Carrera E Claassen J. Continuous electroencephalography in the medical intensive care unit. Crit Care Med 2009; 37:2051–2056. 19384197 10.1097/CCM.0b013e3181a00604

[33] Pantzaris N-D Platanaki C Tsiotsios K. The use of electroencephalography in patients with sepsis: a review of the literature. J Transl Int Med 2021; 9:12–16. 33850796 10.2478/jtim-2021-0007 PMC8016354

[34] Orhun G Esen F Özcan PE. Neuroimaging findings in sepsis-induced brain dysfunction: association with clinical and laboratory findings. Neurocrit Care 2019; 30:106–117. 30027347 10.1007/s12028-018-0581-1

[35] Suchyta MR Jephson A Hopkins RO. Neurologic changes during critical illness: brain imaging findings and neurobehavioral outcomes. Brain Imaging Behav 2010; 4:22–34. 20503111 10.1007/s11682-009-9082-3

[36] Polito A Eischwald F Maho A-L. Pattern of brain injury in the acute setting of human septic shock. Crit Care 2013; 17:R204. 24047502 10.1186/cc12899 PMC4057119

[37] Sharshar T Carlier R Bernard F. Brain lesions in septic shock: a magnetic resonance imaging study. Intensive Care Med 2007; 33:798–806. 17377766 10.1007/s00134-007-0598-y

[38] Morandi A Gunther ML Vasilevskis EE. Neuroimaging in delirious intensive care unit patients: a preliminary case series report. Psychiatry 2010; 7:28–33. PMC2952644 20941349

[39] Morandi A Rogers BP Gunther ML. The relationship between delirium duration, white matter integrity, and cognitive impairment in intensive care unit survivors as determined by diffusion tensor imaging: the VISIONS prospective cohort magnetic resonance imaging study∗. Crit Care Med 2012; 40:2182–2189. 22584766 10.1097/CCM.0b013e318250acdc PMC3378755

[40] Iwashyna TJ Ely EW Smith DM Langa KM. Long-term cognitive impairment and functional disability among survivors of severe sepsis. JAMA 2010; 304:1787–1794. 20978258 10.1001/jama.2010.1553 PMC3345288

[41] Peters van Ton AM Meijer-van Leijsen EMC Bergkamp MI. Risk of dementia and structural brain changes following nonneurological infections during 9-year follow-up. Crit Care Med 2022; 50:554–564. 34432713 10.1097/CCM.0000000000005313

[42] Devlin JW Skrobik Y Gélinas C. Executive summary: clinical practice guidelines for the prevention and management of pain, agitation/sedation, delirium, immobility, and sleep disruption in adult patients in the ICU. Crit Care Med 2018; 46:1532. 30113371 10.1097/CCM.0000000000003259

[43] Andersen-Ranberg NC Poulsen LM Perner A. Haloperidol for the treatment of delirium in ICU patients. N Engl J Med 2022; [Epub ahead of print]. 10.1056/NEJMoa2211868 36286254

[44] Wibrow B Martinez FE Myers E. Prophylactic melatonin for delirium in intensive care (Pro-MEDIC): a randomized controlled trial. Intensive Care Med 2022; 48:414–425. 35220473 10.1007/s00134-022-06638-9

[45▪▪] Lewis K Alshamsi F Carayannopoulos KL. Dexmedetomidine vs other sedatives in critically ill mechanically ventilated adults: a systematic review and meta-analysis of randomized trials. Intensive Care Med 2022; 48:811–840. 35648198 10.1007/s00134-022-06712-2

[46] Barnes-Daly MA Phillips G Ely EW. Improving hospital survival and reducing brain dysfunction at seven california community hospitals: implementing PAD guidelines via the ABCDEF bundle in 6,064 patients. Crit Care Med 2017; 45:171–178. 27861180 10.1097/CCM.0000000000002149

[47] Pun BT Balas MC Barnes-Daly MA. Caring for critically ill patients with the ABCDEF bundle. Crit Care Med 2019; 47:3–14. 30339549 10.1097/CCM.0000000000003482 PMC6298815

[48] Hsieh SJ Jean Hsieh S Otusanya O. Staged implementation of awakening and breathing, coordination, delirium monitoring and management, and early mobilization bundle improves patient outcomes and reduces hospital costs∗. Crit Care Med 2019; 47:885–893. 30985390 10.1097/CCM.0000000000003765 PMC6579661

[49] Winters BD Eberlein M Leung J. Long-term mortality and quality of life in sepsis: a systematic review. Crit Care Med 2010; 38:1276–1283. 20308885 10.1097/CCM.0b013e3181d8cc1d

[50] Iwashyna TJ Cooke CR Wunsch H Kahn JM. Population burden of long-term survivorship after severe sepsis in older Americans. J Am Geriatr Soc 2012; 60:1070–1077. 22642542 10.1111/j.1532-5415.2012.03989.x PMC3374893

[51] van der Slikke EC An AY Hancock REW Bouma HR. Exploring the pathophysiology of postsepsis syndrome to identify therapeutic opportunities. EBioMedicine 2020; 61:103044. 33039713 10.1016/j.ebiom.2020.103044 PMC7544455

[52] Girard TD Thompson JL Pandharipande PP. Clinical phenotypes of delirium during critical illness and severity of subsequent long-term cognitive impairment: a prospective cohort study. Lancet Respir Med 2018; 6:213–222. 29508705 10.1016/S2213-2600(18)30062-6 PMC6709878

[53] Brummel NE Hughes CG Thompson JL. Inflammation and coagulation during critical illness and long-term cognitive impairment and disability. Am J Respir Crit Care Med 2021; 203:699–706. 33030981 10.1164/rccm.201912-2449OC PMC7958515

[54] Latronico N Nattino G Guarneri B. GiVITI Study Investigators. Validation of the peroneal nerve test to diagnose critical illness polyneuropathy and myopathy in the intensive care unit: the Multicentre Italian CRIMYNE-2 Diagnostic Accuracy Study. F1000Res 2014; 3:127. 25309729 10.12688/f1000research.3933.1 PMC4184363

[55] Hermans G Van Mechelen H Clerckx B. Acute outcomes and 1-year mortality of intensive care unit-acquired weakness. A cohort study and propensity-matched analysis. Am J Respir Crit Care Med 2014; 190:410–420. 24825371 10.1164/rccm.201312-2257OC

[56] Jung B Moury PH Mahul M. Diaphragmatic dysfunction in patients with ICU-acquired weakness and its impact on extubation failure. Intensive Care Med 2016; 42:853–861. 26572511 10.1007/s00134-015-4125-2

[57] Latronico N Hermans G. Preiser J-C Herridge M Azoulay E. Critical illness neuromyopathy: clinical, electrophysiological, and histological diagnosis. Post-intensive care syndrome. New York: Springer International; 2020. 43–59.

[58] Latronico N Bolton CF. Critical illness polyneuropathy and myopathy: a major cause of muscle weakness and paralysis. Lancet Neurol 2011; 10:931–941. 21939902 10.1016/S1474-4422(11)70178-8

[59] Hermans G Clerckx B Vanhullebusch T. Interobserver agreement of medical research council sum-score and handgrip strength in the intensive care unit. Muscle Nerve 2012; 45:18–25. 22190301 10.1002/mus.22219

[60] De Jonghe B Sharshar T Lefaucheur J-P. Paresis acquired in the intensive care unit: a prospective multicenter study. JAMA 2002; 288:2859–2867. 12472328 10.1001/jama.288.22.2859

[61] Vanhoutte EK Faber CG van Nes SI. Modifying the Medical Research Council grading system through Rasch analyses. Brain 2012; 135:1639–1649. 22189568 10.1093/brain/awr318 PMC3338921

[62] Hough CL Lieu BK Caldwell ES. Manual muscle strength testing of critically ill patients: feasibility and interobserver agreement. Crit Care 2011; 15:R43. 21276225 10.1186/cc10005 PMC3221972

[63] Fan E Ciesla ND Truong AD. Inter-rater reliability of manual muscle strength testing in ICU survivors and simulated patients. Intensive Care Med 2010; 36:1038–1043. 20213068 10.1007/s00134-010-1796-6 PMC2891143

[64] Ali NA O’Brien JM Jr Hoffmann SP. Acquired weakness, handgrip strength, and mortality in critically ill patients. Am J Respir Crit Care Med 2008; 178:261–268. 18511703 10.1164/rccm.200712-1829OC

[65] Parry SM Berney S Granger CL. A new two-tier strength assessment approach to the diagnosis of weakness in intensive care: an observational study. Crit Care 2015; 19:52. 25882719 10.1186/s13054-015-0780-5 PMC4344764

[66] Latronico N Bertolini G Guarneri B. Simplified electrophysiological evaluation of peripheral nerves in critically ill patients: the Italian multicentre CRIMYNE study. Crit Care 2007; 11:R11. 17254336 10.1186/cc5671 PMC2151880

[67] Rich MM Bird SJ Raps EC. Direct muscle stimulation in acute quadriplegic myopathy. Muscle Nerve 1997; 20:665–673. 9149072 10.1002/(sici)1097-4598(199706)20:6<665::aid-mus2>3.0.co;2-6

[68] Koch S Spuler S Deja M. Critical illness myopathy is frequent: accompanying neuropathy protracts ICU discharge. J Neurol Neurosurg Psychiatry 2011; 82:287–293. 20802220 10.1136/jnnp.2009.192997

[69] Stevens RD Marshall SA Cornblath DR. A framework for diagnosing and classifying intensive care unit-acquired weakness. Crit Care Med 2009; 37:S299–S308. 20046114 10.1097/CCM.0b013e3181b6ef67

[70] Visser LH. Critical illness polyneuropathy and myopathy: clinical features, risk factors and prognosis. Eur J Neurol 2006; 13:1203–1212. 17038033 10.1111/j.1468-1331.2006.01498.x

[71] Latronico N Fenzi F Recupero D. Critical illness myopathy and neuropathy. Lancet 1996; 347:1579–1582. 8667865 10.1016/s0140-6736(96)91074-0

[72] Kelmenson DA Quan D Nordon-Craft A. Electrophysiological abnormalities can differentiate prehospital discharge functional status in critically ill patients with normal strength. Intensive Care Med 2016; 42:1504–1505. 27334267 10.1007/s00134-016-4425-1 PMC4992460

[73] Hermans G Van Mechelen H Bruyninckx F. Predictive value for weakness and 1-year mortality of screening electrophysiology tests in the ICU. Intensive Care Med 2015; 41:2138–2148. 26266842 10.1007/s00134-015-3979-7

[74] Van Aerde N Meersseman P Debaveye Y. Five-year impact of ICU-acquired neuromuscular complications: a prospective, observational study. Intensive Care Med 2020; 46:1184–1193. 31970446 10.1007/s00134-020-05927-5

[75] Piva S Fagoni N Latronico N. Intensive care unit-acquired weakness: unanswered questions and targets for future research: [version 1; peer review: 3 approved]. F1000Res 2019; 8:F1000 Faculty Rev-508. 10.12688/f1000research.17376.1 PMC6480958 31069055

[76] Schweickert WD Pohlman MC Pohlman AS. Early physical and occupational therapy in mechanically ventilated, critically ill patients: a randomised controlled trial. Lancet 2009; 373:1874–1882. 19446324 10.1016/S0140-6736(09)60658-9 PMC9906655

[77] Burtin C Clerckx B Robbeets C. Early exercise in critically ill patients enhances short-term functional recovery. Crit Care Med 2009; 37:2499–2505. 19623052 10.1097/CCM.0b013e3181a38937

[78] Morris PE Goad A Thompson C. Early intensive care unit mobility therapy in the treatment of acute respiratory failure. Crit Care Med 2008; 36:2238–2243. 18596631 10.1097/CCM.0b013e318180b90e

[79] Fuke R Hifumi T Kondo Y. Early rehabilitation to prevent postintensive care syndrome in patients with critical illness: a systematic review and meta-analysis. BMJ Open 2018; 8:e019998. 10.1136/bmjopen-2017-019998 PMC5942437 29730622

[80] Tipping CJ Harrold M Holland A. The effects of active mobilisation and rehabilitation in ICU on mortality and function: a systematic review. Intensive Care Med 2017; 43:171–183. 27864615 10.1007/s00134-016-4612-0

[81] Denehy L Skinner EH Edbrooke L. Exercise rehabilitation for patients with critical illness: a randomized controlled trial with 12 months of follow-up. Crit Care 2013; 17:R156. 23883525 10.1186/cc12835 PMC4056792

[82] Moss M Nordon-Craft A Malone D. A randomized trial of an intensive physical therapy program for patients with acute respiratory failure. Am J Respir Crit Care Med 2016; 193:1101–1110. 26651376 10.1164/rccm.201505-1039OC PMC4872662

[83] Okada Y Unoki T Matsuishi Y. Early versus delayed mobilization for in-hospital mortality and health-related quality of life among critically ill patients: a systematic review and meta-analysis. J Intensive Care Med 2019; 7:57. 10.1186/s40560-019-0413-1 PMC6902574 31867111

[84▪] Menges D Seiler B Tomonaga Y. Systematic early versus late mobilization or standard early mobilization in mechanically ventilated adult ICU patients: systematic review and meta-analysis. Crit Care 2021; 25:16. 33407707 10.1186/s13054-020-03446-9 PMC7789482

[85▪▪] Hodgson CL Bailey M. TEAM Study Investigators and the ANZICS Clinical Trials Group. Early active mobilization during mechanical ventilation in the ICU. N Engl J Med 2022; [Epub ahead of print]. 10.1056/NEJMoa2209083 36286256

[86] Hermans G Wilmer A Meersseman W. Impact of intensive insulin therapy on neuromuscular complications and ventilator dependency in the medical intensive care unit. Am J Respir Crit Care Med 2007; 175:480–489. 17138955 10.1164/rccm.200605-665OC

[87] Van den Berghe G Schoonheydt K Becx P. Insulin therapy protects the central and peripheral nervous system of intensive care patients. Neurology 2005; 64:1348–1353. 15851721 10.1212/01.WNL.0000158442.08857.FC

[88] Finfer S Chittock DR. NICE-SUGAR Study Investigators. Intensive versus conventional glucose control in critically ill patients. N Engl J Med 2009; 360:1283–1297. 19318384 10.1056/NEJMoa0810625

[89] Vanhorebeek I Latronico N Van den Berghe G. ICU-acquired weakness. Intensive Care Med 2020; 46:637–653. 32076765 10.1007/s00134-020-05944-4 PMC7224132

[90] Puthucheary ZA Rawal J McPhail M. Acute skeletal muscle wasting in critical illness. JAMA 2013; 310:1591–1600. 24108501 10.1001/jama.2013.278481

[91] Rugeles S Villarraga-Angulo LG Ariza-Gutiérrez A. High-protein hypocaloric vs normocaloric enteral nutrition in critically ill patients: a randomized clinical trial. J Crit Care 2016; 35:110–114. 27481744 10.1016/j.jcrc.2016.05.004

[92] Hermans G Casaer MP Clerckx B. Effect of tolerating macronutrient deficit on the development of intensive-care unit acquired weakness: a subanalysis of the EPaNIC trial. Lancet Respir Med 2013; 1:621–629. 24461665 10.1016/S2213-2600(13)70183-8

[93▪▪] Chapple L-AS Kouw IWK Summers MJ. Muscle protein synthesis after protein administration in critical illness. Am J Respir Crit Care Med 2022; 206:740–749. 35584344 10.1164/rccm.202112-2780OC

[94] Bolton CF. Critical illness polyneuropathy: a useful concept. Muscle Nerve 1999; 22:419–422. 10086906 10.1002/(sici)1097-4598(199903)22:3<419::aid-mus18>3.0.co;2-y

[95] Herridge MS Tansey CM Matté A. Functional disability 5 years after acute respiratory distress syndrome. N Engl J Med 2011; 364:1293–1304. 21470008 10.1056/NEJMoa1011802

[96] Herridge MS Cheung AM Tansey CM. One-year outcomes in survivors of the acute respiratory distress syndrome. N Engl J Med 2003; 348:683–693. 12594312 10.1056/NEJMoa022450

[97] Fan E Dowdy DW Colantuoni E. Physical complications in acute lung injury survivors: a two-year longitudinal prospective study. Crit Care Med 2014; 42:849–859. 24247473 10.1097/CCM.0000000000000040 PMC3959239

[98] Needham DM Dinglas VD Bienvenu OJ. NIH NHLBI ARDS Network. One year outcomes in patients with acute lung injury randomised to initial trophic or full enteral feeding: prospective follow-up of EDEN randomised trial. BMJ 2013; 346:f1532. 23512759 10.1136/bmj.f1532 PMC3601941

[99] Latronico N Peli E Calza S. Physical, cognitive and mental health outcomes in 1-year survivors of COVID-19-associated ARDS. Thorax 2022; 77:300–303. 34588274 10.1136/thoraxjnl-2021-218064

[100] Latronico N Herridge MS. Unraveling the myriad contributors to persistent diminished exercise capacity after critical illness. Intensive Care Med 2015; 41:1854–1856. 26160731 10.1007/s00134-015-3966-z

[101] Guarneri B Bertolini G Latronico N. Long-term outcome in patients with critical illness myopathy or neuropathy: the Italian multicentre CRIMYNE study. J Neurol Neurosurg Psychiatry 2008; 79:838–841. 18339730 10.1136/jnnp.2007.142430

[102] Koch S Wollersheim T Bierbrauer J. Long-term recovery In critical illness myopathy is complete, contrary to polyneuropathy. Muscle Nerve 2014; 50:431–436. 24415656 10.1002/mus.24175

[103] Hopkins RO Weaver LK Collingridge D. Two-year cognitive, emotional, and quality-of-life outcomes in acute respiratory distress syndrome. Am J Respir Crit Care Med 2005; 171:340–347. 15542793 10.1164/rccm.200406-763OC

[104] Kamdar BB Huang M Dinglas VD. National Heart, Lung, and Blood Institute Acute Respiratory Distress Syndrome Network. Joblessness and lost earnings after acute respiratory distress syndrome in a 1-year national multicenter study. Am J Respir Crit Care Med 2017; 196:1012–1020. 28448162 10.1164/rccm.201611-2327OC PMC5649982

[105] Su H Thompson HJ May S. Association of job characteristics and functional impairments on return to work after ARDS. Chest 2021; 160:509–518. 33727035 10.1016/j.chest.2021.03.008 PMC8411444

[106] Rudd KE Johnson SC Agesa KM. Global, regional, and national sepsis incidence and mortality, 1990–2017: analysis for the Global Burden of Disease Study. Lancet 2020; 395:200–211. 31954465 10.1016/S0140-6736(19)32989-7 PMC6970225

